# 3D printing for surgical planning of canine oral and maxillofacial surgeries

**DOI:** 10.1186/s41205-022-00142-y

**Published:** 2022-06-09

**Authors:** Yu-Hui Huang, Bonnie Lee, Jeffrey A. Chuy, Stephanie L. Goldschmidt

**Affiliations:** 1 Department of Radiology, University of Minnesota, 420 Delaware Street SE, Minneapolis, MN 55455 USA; 2 Department of Radiology, Minneapolis VA Medical Center, 1 Veterans Dr, Minneapolis, MN 55417 USA; 3 College of Veterinary Medicine, University of Minnesota, 1352 Boyd Ave, St Paul, MN 55108 USA

**Keywords:** 3D printing, Surgical planning, Veterinary oral and maxillofacial surgery, Desktop vat polymerization, Stereolithography

## Abstract

**Background:**

Advanced diagnostic imaging is an essential part of preoperative planning for oral and maxillofacial surgery in veterinary patients. 3-dimensional (3D) printed models and surgical guides generated from diagnostic imaging can provide a deeper understanding of the complex maxillofacial anatomy, including relevant spatial relationships. Additionally, patient-specific 3D printed models allow surgeons and trainees to better examine anatomical features through tactile and visuospatial feedback allowing for improved preoperative planning, intraoperative guidance, and enhanced trainee education. Furthermore, these models facilitate discussions with pet owners, allowing for improved owner understanding of pathology, and educated decision-making regarding treatment.

**Case presentation:**

Our case series consists of three 3D printed models segmented from computed tomography (CT) and cone beam CT (CBCT) and fabricated via desktop vat polymerization for preoperative planning and intraoperative guidance for resection of maxillary osteosarcoma, mandibular reconstruction after mandibulectomy, and gap arthroplasty for temporomandibular joint ankylosis in dogs.

**Conclusions:**

We illustrate multiple benefits and indications for 3D printing in veterinary oral and maxillofacial surgery. 3D printed models facilitate the understanding of complex surgical anatomy, creating an opportunity to assess the spatial relationship of the relevant structures. It facilitates individualized surgical planning by allowing surgeons to tailor and augment the surgical plan by examining patient-specific anatomy and pathology. Surgical steps may also be simulated in advance, including planning of osteotomy lines, and pre-contouring of titanium plates for reconstruction. Additionally, a 3D printed model and surgical guide also serve as invaluable intraoperative reference and guidance. Furthermore, 3D printed models have the potential to improve veterinary resident and student training as well as pet owner understanding and communication regarding the condition of their pets, treatment plan and intended outcomes.

## Background

The field of veterinary oral and maxillofacial surgery is evolving thanks to innovation in surgical treatment options, increased willingness of pet owners to pursue advanced surgical care for their pets, and providers striving to match the standard of care established in human medicine [1]. Like its human counterpart, veterinary maxillofacial surgery involves complex and delicate craniofacial anatomy. Advanced diagnostic imaging, specifically computed tomography (CT) and cone beam computed tomography (CBCT), play an essential role in preoperative planning to evaluate the extent of disease in malignancy or injuries in trauma [2]. However, conventional CT and CBCT remain limited in portraying spatial relationships for complex anatomic details; even with 3-dimensional (3D) CT scan reconstruction, these imaging modalities lack the freedom of tactile manipulation. These shortcomings can be overcome by translating conventional imaging into a 3D printed model. With advances and increased accessibility of the 3D printing technology, there has been increased adoption of medical 3D printing including in veterinary medicine [3, 4]. Desktop vat polymerization is one of the leading technologies that has made 3D printing more accessible and affordable. The modality uses smaller printers and thus minimizes the space requirement while maintaining the high resolution needed for accurate fabrication of the complex and delicate maxillofacial anatomy. The 3D printed patient specific models allow surgeons, trainees, and pet owners to develop a better understanding of the anatomical features; this enhanced understanding further translates to improved preoperative planning and intraoperative guidance [5]. The application and benefits of 3D printing in oral maxillofacial surgery in humans have already been demonstrated [6] with increasing adoption in veterinary medicine [4, 7].

The present case series consist of canine skull models segmented from CT and CBCT. With carefully validated workflows, the process of 3D printing anatomic models from medical imaging data of CT and CBCT has been shown to have inaccuracies limited to less than 1 mm [8]. The imaging files were downloaded as Digital Imaging and Communications (DICOM) files and imported into FDA-cleared DICOM to PRINT® (D2P) (3D Systems, Inc. Rock Hill, SC) for segmentation to construct the 3D models. The files were then exported as Standard Tessellation Language (STL) files, imported into printer’s proprietary software PreForm v3.21.0 (Formlabs Inc. Somerville, MA), and 3D printed using Formlabs Form 3B desktop 3D printer (Formlabs Inc. Somerville, MA). The 3D printed models were used for preoperative planning and intraoperative guidance for resection of maxillary osteosarcoma, mandibular reconstruction after mandibulectomy, and gap arthroplasty for temporomandibular joint ankylosis in dogs.

## Case presentation

### Case 1

**Fig. 1:**
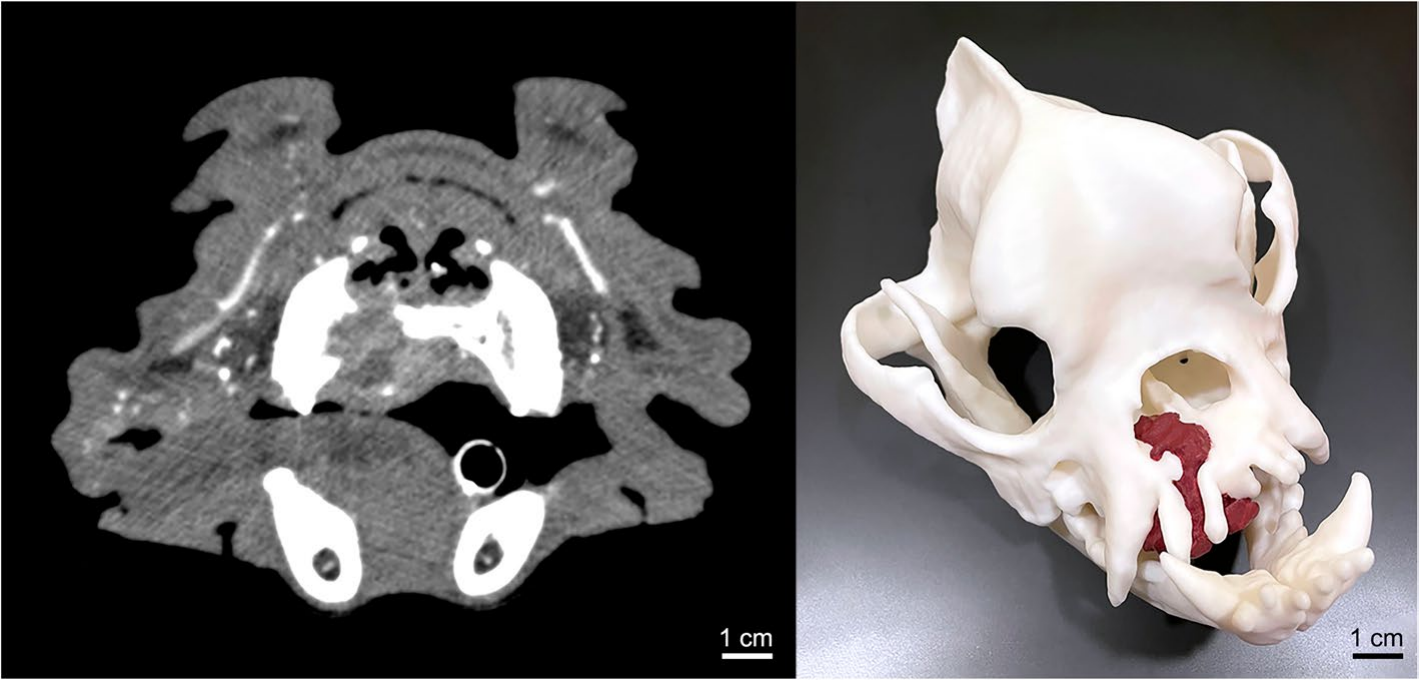
**A** Transverse section of preoperative contrast-enhanced CT demonstrating early right nasal invasion of the right maxillary osteosarcoma. **B** 3D printed canine skull in white resin with right maxillary chondroblastic osteosarcoma painted in red

Patient C.G. is a 9-year-old male English bulldog who presented with decreased oral intake and oral bleeding secondary to biopsy confirmed maxillary chondroblastic osteosarcoma. A preoperative contrast-enhanced CT (Toshiba Acquilion 64 CFX, Toshiba Medical Systems, Tustin, CA) was obtained with 1.0 mm slice thickness revealing localized osteolysis and early right nasal invasion without evidence of thoracic metastatic disease (Fig. 1A). The skull and tumor took approximately 4 h to segment, 37 h and 25 min to print, and used 352 ml of white resin. A 1:1 skull model with tumor was printed in white resin with the tumor painted in red to contrast against the white skull by a radiology resident using the CT data as reference (Fig. 1B). The 3D printed model was utilized for preoperative planning and sterilized to serve as intraoperative reference for a bilateral rostral maxillectomy. Visualization and tactile assessment of tumor extent in the 3D printed model led to the conclusion that curative intent surgery with 2 cm margins would carry a high risk of potential morbidity. Thus, it was elected to excise the tumor with conservative margins (0.5–1 cm) and treat with postoperative radiation therapy. Adjuvant radiation was performed (20 × 2.8 Gy) to a total dose of 56 Gy using 6 MV photons and an intensity-modulated radiation therapy (IMRT) plan with image-guided radiation therapy (IGRT); the protocol was adapted to reduce the dose per fraction to limit the likelihood of late toxicity while maintaining good tumor control [9]. C.G. developed a 15 × 15 mm oronasal fistula secondary to postoperative dehiscence and underwent subsequent repair following the completion of radiation therapy. A buccal mucosal advancement flap was utilized for closure of the oronasal defect. Majority of the closure healed within 14 days. However, there was a small area of dehiscence and a resultant defect of approximately 5 mm. As the patient was asymptomatic, no further repair was performed. At the time of this writing, the patient is doing well in remission and is approximately 11 months post-op.

### Case 2

**Fig. 2:**
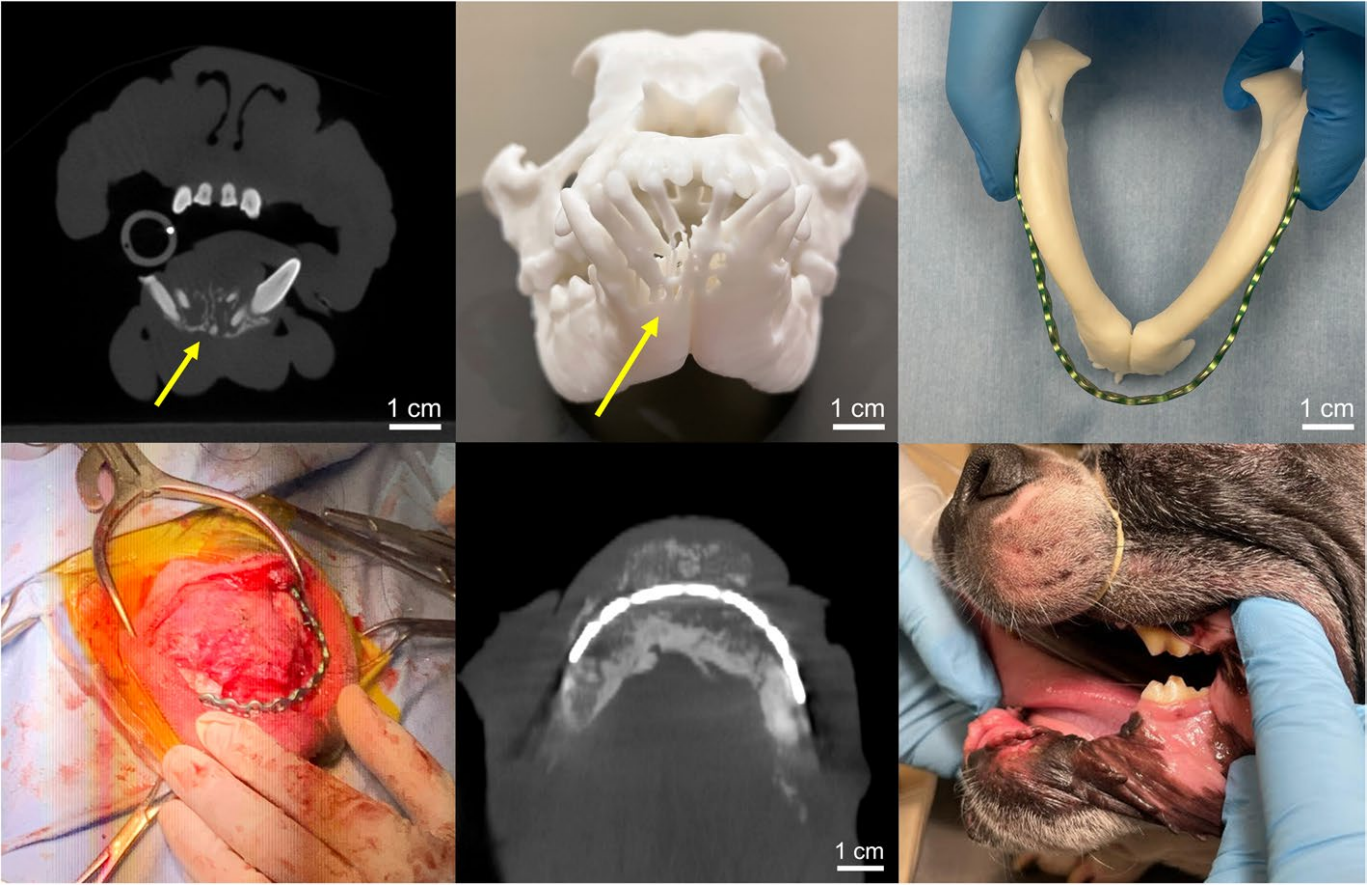
**A** Transverse section of preoperative CBCT demonstrating extensive osteolysis denoted by the yellow arrow of rostral mandible secondary to oral plasma cell tumor. **B** 3D printed canine skull in white resin demonstrating the mandibular osteolysis by the yellow arrow. **C** Pre-contouring of titanium plate for mandibular reconstruction using the 3D printed mandible. **D** Intraoperative photograph of the mandibular reconstruction with pre-contoured plate and bone morphogenetic protein (BMP) grafting. **E** 4 months follow-up CBCT demonstrates mandibular bony consolidation. **F** 4 months follow-up photograph demonstrates well healed reconstructed mandible

Patient P.P. is an 8-year-old male Boston terrier who presented with an oral plasma cell tumor of his rostral mandible. Despite the presence of extensive osteolysis, the dog was asymptomatic at presentation. Preoperative CBCT was obtained with 0.9 mm slice thickness (Xoran Vet Cat, Xoran Technologies, Ann Arbor, MI) (Fig. 2A). Subsequent chest radiograph demonstrated normal thorax without evidence of pulmonary metastasis. The skull took approximately 2 h to segment, 23 h and 27 min to print, and used 176 ml of white resin. A 1:1 skull model was printed in white resin (Fig. 2B). The 3D printed skull model was utilized for preoperative planning and subsequently sterilized to serve as an intraoperative reference for bilateral rostral mandibulectomy and pre-contouring of the titanium plate for subsequent mandibular reconstruction (Fig. 2C). Surgical margins were planned 1 cm from the area of bone lysis documented on CT scan. To obtain this margin, a rostral mandibulectomy to the distal aspect of the mandibular fourth premolars bilaterally (308, 408) was performed. Post-operative radiographs revealed that all tooth material was removed, and there was no evidence of abnormal bone in the remaining mandible. Plating was performed 6 weeks post-operatively at the time of bone morphogenetic protein (BMP) grafting to allow the intraoral incisions to completely heal and minimize oral bacterial contamination of the plate. The printed model saved approximately 30 min of operation and anesthesia time by allowing pre-contouring of the titanium plate for the mandibular reconstruction. Patient is doing well post-operatively with bony consolidation demonstrated on 4 months follow-up CBCT (Fig. 2E-F) and is approximately 8 months post-op.

### Case 3

**Fig. 3:**
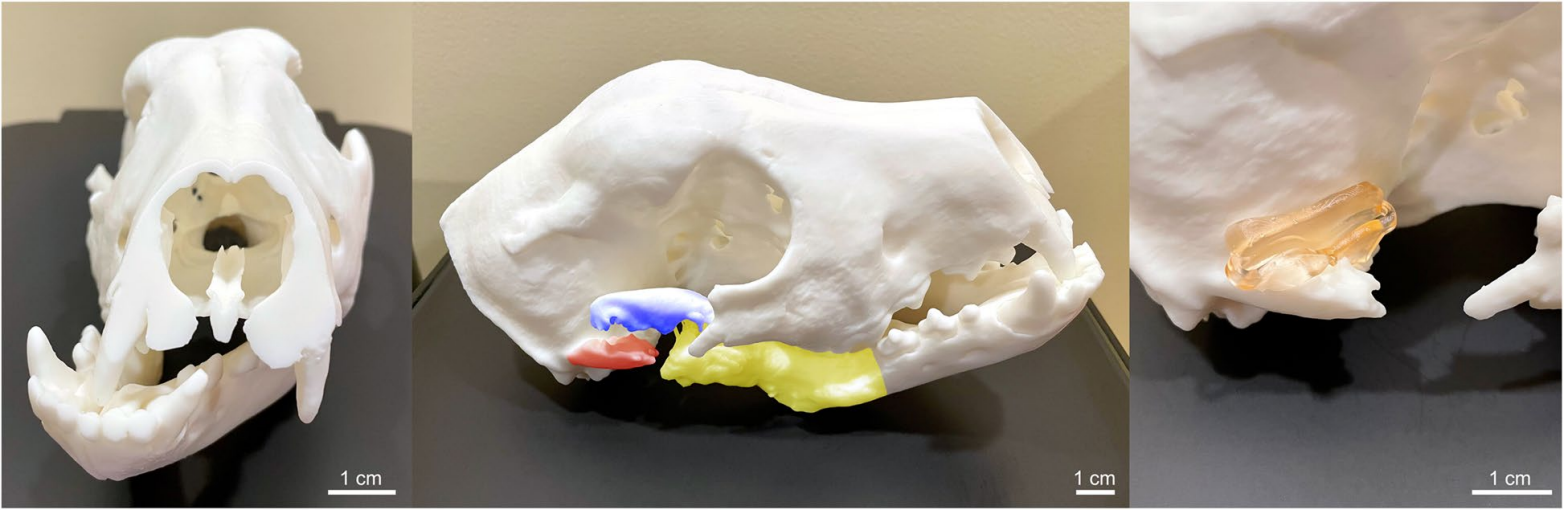
**A** Frontal view of the 3D printed canine skull printed in white resin. **B** Lateral view of the 3D printed skull demonstrating the right temporomandibular joint ankylosis with heterotopic temporal (red), zygomatic (blue), and mandibular bone (yellow). **C** Cutting guide printed in surgical resin and seated on the heterotopic temporal bone of the 3D printed cranium

**Fig. 4:**
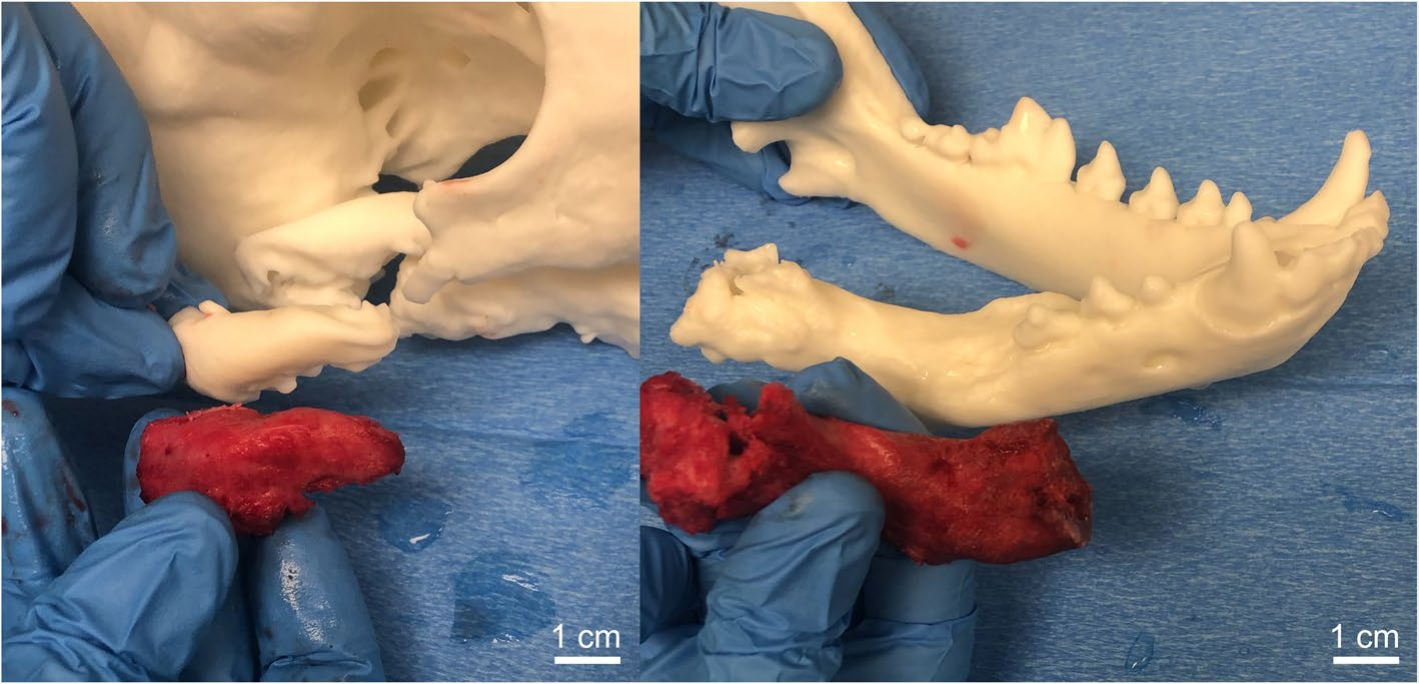
**A** Osteotomy of the heterotopic zygomatic bone with reference to the 3D printed model. **B** Osteotomy of heterotopic right caudal mandible with reference to the 3D printed mandible

Patient C.B. is a 6-month-old male silver Labrador retriever who presented with severely decreased range of motion secondary to recurrent temporomandibular joint (TMJ) ankylosis post gap arthroplasty. Joint ankylosis developed secondary to a dog bite that occurred at 8 weeks of age. Initial gap arthroplasty was performed 10 weeks after the trauma due to progressive decrease in range of motion with eventual inability to open mouth. Approximately 3 months following the initial surgery, the range of motion began to progressively decrease and it was suspected that there was new bone formation with subsequent ankylosis. CBCT was obtained with 0.9 mm slice thickness (Xoran Vet Cat, Xoran Technologies, Ann Arbor, MI) confirming TMJ ankylosis. Due to the increased complexity of the surgery, 3D models with surgical guides were printed. The skull took approximately 1.5 h to segment, 25 h to print, and used 286 ml of white resin. The surgical guides took 3.5 h to design, 2 h 11 min to print, and used 5 ml of surgical guide resin. A 1:1 skull model was printed in white resin (Fig. 3A and B) and a surgical guide was fabricated for the osteotomy of the heterotopic temporal bone; the surgical guide was printed in biocompatible surgical guide resin (Fig. 3C). The 3D printed skull and surgical guide were sterilized to serve as intraoperative reference and guidance for the gap arthroplasty and osteotomies of the heterotopic temporal, zygomatic and mandibular bone (Fig. 4A and B). However, due to anatomical changes in the 2.5 week interval between CT scan and surgery, the surgical guide no longer fits perfectly intra-operatively, highlighting the need to minimize the time between the initial preoperative CT scan for guide design and surgery especially in a juvenile patient with TMJ ankylosis. Regardless, the 3D printed models and guide saved up to an hour of operation and anesthesia time by allowing the surgeon and trainee to have a tactile appreciation of the lesion and thus be more confident in operating in a very high risk location. The patient currently maintains a normal range of motion 5 months postoperatively.

## Discussion

Our case series demonstrates potential benefits and indications for 3D printing in veterinary oral and maxillofacial surgery by improving the diagnosis and treatment of pathology through more accurate preoperative planning. Veterinary medicine faces a unique challenge where canine skull shape can range from brachycephalic with short and wide cranial proportions as seen in breeds such as English bulldog in Case 1 and Boston terrier in Case 2 to mesaticephalic with medium muzzle length and intermediate cranial proportions in breeds such as Labrador retriever in Case 3, and dolichocephalic with long and narrow cranial proportions and long muzzle [10]. Our 3D printed skull in Case 1 with maxillary osteosarcoma enhanced both the surgeon’s and pet owner’s understanding of the tumor extent leading to the augmentation of treatment plan from curative intent surgery with wide margin to conservative margin with postoperative radiation therapy in order to reduce the potential high risk of morbidity with wide margin. Traditional 2D images of CT or CBCT can be challenging for pet owners to interpret; allowing the owners to see and physically manipulate the 3D model of their pet facilitated comprehension of the surgical complexity, possible complications, and enriched the informed consent process resulting in proceeding with the conservative margin surgery followed by radiation therapy as in Case 1.

Patient-specific 3D printed models can also facilitate complex surgical planning with the ability to simulate the surgical steps in advance including planning of osteotomy lines and pre-contouring of titanium plates for reconstruction. The fabrication of custom-fitting surgical guides for osteotomies further translates the virtual preoperative plan to surgery by improving the precision and accuracy for optimal postoperative result [11]. These benefits have been shown to reduce surgical time and cost [12, 13]. Winer et al. reported saving at least 15 min of intraoperative time using 3D printed skulls for pre-contouring reconstruction plates in 19 veterinary patients [4]. The printed models saved approximately 30 min of operation time for our mandibular reconstruction and up to an hour for the TMJ ankylosis case by allowing the surgeon and trainee to have a tactile appreciation of the lesion and thus improve surgical technique and confidence. The complication risk in canine oral surgery, specifically with mandibulectomy and maxillectomy, is increased by 36% for each additional hour of surgery, where the reported complications include hemorrhage, aspiration pneumonia, incisional dehiscence, mandibular drift and malocclusion, oronasal fistula formation, and death [14]. Thus, minimizing surgical time with improved preoperative planning and intraoperative guidance from 3D printed models and surgical guides has the potential to improve patient outcomes.

In addition to preoperative planning and intraoperative guidance, 3D printed models may enhance veterinary trainee education. Preece et al. demonstrate that students who used 3D models performed better and had a better learning experience than those using digital models or textbooks suggesting that 3D models enhance understanding of anatomical structures and their relationships [15]. Patient specific 3D printed models further facilitate trainee’s understanding of the complex surgical anatomy, potentially reducing the need for cadaveric specimens as well as morbidity and mortality for patients during their surgical experience on living patients [5]. Incorporation of 3D printing in training of surgeons is well documented including a program at the University of North Carolina at Chapel Hill that fabricates patient specific models to train surgical residents [16].

Most barriers and limitations of 3D printed models include the upfront cost of acquiring a 3D printer, materials, segmentation software, the expertise required for the fabrication process and quality assurance, and the time needed for printing and post-processing. However, 3D printing has become more accessible with desktop vat polymerization technology making it more affordable with a smaller footprint printer minimizing the space requirement while maintaining the high resolution needed for accurate fabrication of the complex delicate maxillofacial anatomy. The multitude of biocompatible materials from Formlabs Surgical Guide Resin to BioMed Resins that can be sterilized for intraoperative patient contact use will likely facilitate further adoption of 3D printing in the clinical and surgical setting as it has done for us. Depending on the size and complexity of the canine skull, it took between 23 to 37 h to complete the printing process for our case series and approximately 2 additional hours for post-processing. However, the 3D printing and post-processing time required did not negatively impact patient care in the cases highlighted in this series. In all three cases, the printing and processing occurred during pre-surgical planning. As such, in general, we would not expect the time commitment required to complete the 3D printing process to affect patient care as the candidates selected for 3D printing are usually complex cases that require preoperative imaging acquisition followed by a stage procedure for thorough diagnosis and preoperative planning. Thus, the 3D printing process takes place concurrently with the preoperative surgical planning prior to the scheduled surgery. However, as demonstrated in Case 3, changes in patient anatomy occurred between initial CT to the time of surgery, where the surgical guide no longer fits perfectly intra-operatively, highlighting the need to minimize time between the CT acquisition for guide design and surgery especially in juvenile patients. With advancement of 3D printing technologies, production time and cost will likely continue to decrease.

## Conclusion

The 3D printed models altered the surgical plan in Case 1 to minimize potential complications, expedited the pre-contouring of titanium plate for mandibular reconstruction in Case 2, and ensured precision of planned osteotomies in Case 3 to avoid inadvertently violating the cranium while saving up to an hour of operation time. We demonstrated that 3D printed models can improve preoperative planning and intraoperative guidance while enhancing veterinary training and pet owner communication. As 3D printing technology continues to advance, there will be increased adoption in veterinary medicine as it is already evidenced in human medicine with more hospitals offering medical 3D printing and approved Current Procedural Terminology (CPT) codes. We aim to continually refine and validate our workflow while collecting more outcome-based evidence on the utilization of medical 3D printing for our veterinarian patients with complex oral and maxillofacial needs.

## Data Availability

The datasets used in the current study are available from the corresponding author on reasonable request.

## Abbreviations

3D: 3-Dimensional
CT: Computed tomography
CBCT: Cone beam computed tomography
DICOM: Digital Imaging and Communications
STL: Standard Tessellation Language
IMRT: Intensity-modulated radiation therapy
IGRT: Image-guided radiation therapy
TMJ: Temporomandibular joint
BMP: Bone morphogenetic protein
CPT: Current Procedural Terminology
